# Characterization of the cytokinin-responsive transcriptome in rice

**DOI:** 10.1186/s12870-016-0932-z

**Published:** 2016-12-08

**Authors:** Tracy Raines, Ivory C. Blakley, Yu-Chang Tsai, Jennifer M. Worthen, José Manuel Franco-Zorrilla, Roberto Solano, G. Eric Schaller, Ann E. Loraine, Joseph J. Kieber

**Affiliations:** 1 Department of Biology, University of North Carolina, Chapel Hill, NC 27599-3280 USA; 2 Department of Bioinformatics and Genomics, University of North Carolina at Charlotte, North Carolina Research Campus, Kannapolis, NC 28081 USA; 3 Department of Biological Sciences, Dartmouth College, Hanover, NH 03755 USA; 4 Genomics Unit, Centro Nacional de Biotecnología (CNB)-Consejo Superior de Investigaciones Científicas (CSIC), Darwin 3, 28049 Madrid, Spain; 5 Department of Plant Molecular Genetics, Centro Nacional de Biotecnología (CNB)-Consejo Superior de Investigaciones Científicas (CSIC), Darwin 3, 28049 Madrid, Spain; 6 Present address: AgBiome, Inc., 104 TW Alexander Drive, Bldg 18, Research Triangle Park, NC 27713 USA; 7 Present address: Department of Agronomy, National Taiwan University, Taipei, 10617 Taiwan

**Keywords:** Rice, Arabidopsis, Plant hormone, Cytokinin, Transcriptomics

## Abstract

**Background:**

Cytokinin activates transcriptional cascades important for development and the responses to biotic and abiotic stresses. Most of what is known regarding cytokinin-regulated gene expression comes from studies of the dicotyledonous plant *Arabidopsis thaliana*. To expand the understanding of the cytokinin-regulated transcriptome, we employed RNA-Seq to analyze gene expression in response to cytokinin in roots and shoots of the monocotyledonous plant rice.

**Results:**

We identified over 4,600 and approximately 2,400 genes differentially expressed in response to cytokinin in roots and shoots respectively. There were some similarities in the sets of cytokinin-regulated genes identified in rice and Arabidopsis, including an up-regulation of genes that act to reduce cytokinin function. Consistent with this, we found that the preferred DNA-binding motif of a rice type-B response regulator is similar to those from Arabidopsis. Analysis of the genes regulated by cytokinin in rice revealed a large number of transcription factors, receptor-like kinases, and genes involved in protein degradation, as well as genes involved in development and the response to biotic stress. Consistent with the over-representation of genes involved in biotic stress, there is a substantial overlap in the genes regulated by cytokinin and those differentially expressed in response to pathogen infection, suggesting that cytokinin plays an integral role in the transcriptional response to pathogens in rice, including the induction of a large number of WRKY transcription factors.

**Conclusions:**

These results begin to unravel the complex gene regulation after cytokinin perception in a crop of agricultural importance and provide insight into the processes and responses modulated by cytokinin in monocots.

**Electronic supplementary material:**

The online version of this article (doi:10.1186/s12870-016-0932-z) contains supplementary material, which is available to authorized users.

## Background

Phytohormones regulate many aspects of plant growth and development as well as responses to the environment in part through an integrated modulation of the transcriptome [1]. Cytokinins are *N* ^6^-substituted adenine derivatives that were discovered based on their ability to promote cell division in cultured cells [2, 3]. Since their discovery, cytokinins have been implicated in almost all facets of plant growth and development, including leaf senescence, meristem maintenance, sink/source relationships, and biotic/abiotic interactions [4–6]. Much progress has been made in understanding cytokinin biosynthesis, signaling, and gene regulation, mostly from genetic and molecular studies in the dicot *Arabidopsis thaliana*, including the identification of the key elements of the cytokinin signaling pathway [6], which enabled studies of the function of cytokinin in other plants, including its role in nodulation in legumes [7] and phylotaxy in maize [8].

The cytokinin signaling pathway is comprised of a ‘two-component’ phosphorelay that culminates in the activation of the type-B response regulators (RRs), transcription factors that modulate downstream gene expression [6]. The first step in the signaling pathway is mediated by the hybrid histidine-kinase receptors (HKs), which are embedded in the endoplasmic reticulum membrane such that the amino-terminal cytokinin-binding domain is localized within the lumen of the endoplasmic reticulum [9, 10]. Upon cytokinin binding, the HKs autophosphorylate on a conserved histidine, and then transfer the phosphate to an Asp residue within their C-terminal receiver domain [11–14]. This phosphate is subsequently transferred to the histidine phosphotransfer proteins (HPs), which in turn transfer the phosphate to the type-A and type-B RRs [15, 16]. The type-B RRs are activated by phosphorylation on a conserved Asp and act as positive elements of signaling. They contain a C-terminal DNA-binding domain and initiate a complex transcriptional cascade to drive the appropriate gene expression changes downstream of cytokinin perception [17–20]. The type-A *RR*s are cytokinin primary response genes that negatively regulate the signaling pathway [21–24].

Rice (*Oryza sativa*) is a staple crop for almost half of the world’s population. The global consumption of rice has risen steadily over the past 50 years and is projected to continue to increase as the world’s population rises through 2050 [25], pointing toward an urgent need to increase production. Cytokinin plays a key role in determining yield from rice; a major quantitative trait loci underlying yield was identified as a cytokinin oxidase gene, which encodes a protein that degrades cytokinin [26]. Phylogenetic analysis has identified rice homologs of Arabidopsis cytokinin signaling pathway components [27–29] and functional studies of a few of these genes have confirmed their role in cytokinin signaling. For example, the *OsHK6* gene encodes an HK that binds cytokinin and complements an Arabidopsis cytokinin receptor mutant [30]. Similarly, a rice type-B RR (OsRR22) complements a type-B RR mutant of Arabidopsis [27]. Disruption of expression of *OsHP1* and *OsHP2* by RNAi results in reduced sensitivity to exogenous cytokinin and phenotypes consistent with reduced cytokinin function [31]. Overexpression of several type-A *OsRRs* in rice blocks shoot regeneration in response to cytokinin and results in reduced root sensitivity to cytokinin, suggesting that they negatively regulate cytokinin signaling, similar to what is found in Arabidopsis [32, 33]. While these results indicate that, at a basic level, the backbone of the cytokinin signaling pathway likely operates in a similar manner in monocots and dicots, the downstream processes regulated by cytokinin in rice have yet to be established.

Global gene expression responses to cytokinin have been extensively studied in Arabidopsis using microarray and RNA-Seq analyses [34–38]. Many genes that are differentially expressed as early as 15 min after the treatment encode transcription factors, suggesting that cytokinin not only triggers immediate gene expression changes, but also activates complex transcriptional cascades. Here, we use RNA-Seq to identify genes regulated by cytokinin in the roots and shoots of rice seedlings. Identifying these changes in response to exogenous cytokinin defines the distinct patterns of expression in response to cytokinin in the two different tissues. Comparing the differentially expressed genes in rice to a similar experiment in Arabidopsis reveals similarities and differences in the role of cytokinin between these monocot and dicot species. This study begins to unravel the complex gene regulation after cytokinin perception in a crop of agricultural importance and provides insight into the processes and responses modulated by cytokinin in monocots.

## Results and discussion

### Identification of cytokinin-responsive genes in rice

To investigate cytokinin regulation of gene expression in a monocot, we performed high throughput cDNA sequencing (RNA-Seq) of libraries prepared from rice seedlings treated for two hours with the cytokinin benzyladenine (BA). Rice seedlings were grown hydroponically and cytokinin delivered via addition to the hydroponic media. Twelve libraries were prepared in total, comprised of three replicates each of BA and mock-treated roots and shoots. Libraries were sequenced on the Illumina HiSeq platform, yielding approximately 30 to 50 million single-end, 100-bp reads per library. In each library, at least 90% of reads had a mean Phred score of ≥ 28 and more than 95% could be mapped to a single location in the rice genome. Altogether, more than 447 million reads mapped to a unique genomic location. Thus coverage of the rice transcriptome was deep enough to provide a detailed view of how cytokinin affected gene expression in both roots and shoots of rice seedlings. To facilitate re-use of the data in other studies, we configured the Integrated Genome Browser (IGB) [39] to offer access to RNA-Seq alignment files, pre-computed coverage graphs, and splice junction files. To view the data, readers should download the browser, select the latest rice genome, and then browse and select data in the Data Access tab.

Read alignments were compared to rice gene models from the Michigan State University rice annotation project’s MSU7 release [40]. Comparing read alignments to annotated genes in MSU7 identified approximately 30,000 genes with 20 mapped reads or more across all samples (Additional file 1: Table S1). Using this as a minimal threshold for calling a gene expressed, we detected expression for 53% of the 55,987 annotated rice genes. For comparisons between genes, expression values were calculated as the number of reads per kilobase of expressed sequence per million mapped reads (RPKM; Additional file 2: Table S2). Other gene model collections are available, such as annotations from the Rice Annotation Project Database [41], but we chose to use the MSU7 release in part because of the availability of informatics tools needed for functional interpretation of the data, such as GO annotations and Arabidopsis ortholog assignments. In general, we have found that the MSU7 and RGAP-DB annotation collections are congruent in that genes annotated in MSU7 are typically present in the RGAP-DB annotations, and vice versa; tools that map gene names between models are available, and to further facilitate comparisons, we configured IGB to provide both sets of annotations together with the RNA-Seq data.

**Fig. 1:**
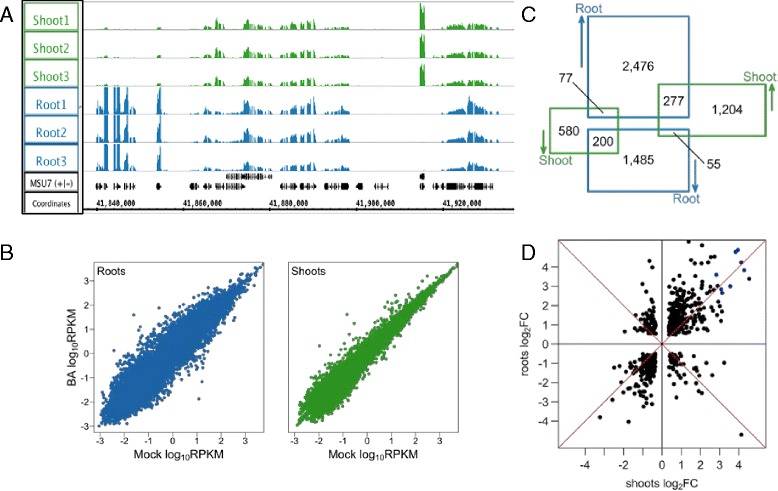
Gene expression in BA-treated and mock-treated rice seedlings. **a** RNA-Seq coverage graphs from chromosome 1 showing mock-treated rice root and shoots. Graphs in the different tracks use the same y-axis scale, and peaks represent regions of high expression. The region in view includes the following genes: 1) LOC_Os01g72170^*DE*^; 2) LOC_Os01g72190; 3) LOC_Os01g72200; 4) LOC_Os01g72205 ^*DE*^; 5) LOC_Os01g72210 ^*DE*^; 6) LOC_Os01g72220; 7) LOC_Os01g72230; 8) LOC_Os01g72240; 9) LOC_Os01g72250; 10) LOC_Os01g72260^*DE*^; 11) LOC_Os01g72270^*DE*^; 12) LOC_Os01g72280^*DE*^; 13) LOC_Os01g72290^*DE*^; where (^*DE*^) indicates genes that are differentially expressed between roots and shoots. **b** Expression in BA-treated samples in log_2_ of RPKM compared to mock-treated samples in roots (*left*) and shoots (*right*). **c** Venn diagram showing the number of genes up or down-regulated in roots and shoots. **d** Plot showing log_2_ fold change in roots versus shoots for genes that were differentially regulated by BA treatment in both tissues. Data points in the upper right and lower left quadrants represent genes that were changed in the same direction in both shoots and roots. Blue data points indicate type-A RRs

Many of the same genes were expressed in shoots and roots of rice, but the overall profile of gene expression was different between the two tissues. The similarities and differences between gene expression in roots and shoots is clear when visualized on a chromosome or region-wide scale using IGB. Figure 1a shows an example of RNA-Seq coverage graphs from the six control libraries depicting read density by chromosome position for a portion of chromosome 1. Several of these genes show differential expression in the roots relative to shoot. Such regions are widespread throughout the rice genome. In total, more than 17,000 genes were differentially expressed between the roots and shoots in control conditions (Additional file 3: Table S3), with about 9,000 of them having a higher expression level in the root.

Consistent with RNA-Seq studies in other plant species [37, 42, 43], a relatively small number of highly expressed genes in rice gave rise to most of the sequence reads (Additional file 1: Tables S1 and Additional file 2: Table S2). In each of the 12 libraries, more than 75% of reads mapped to the top 10% of the most highly expressed genes. Overall diversity of gene expression in roots and shoots was similar, but the types of genes that were most highly expressed in the roots versus shoots reflected tissue-specific functions. In shoots, genes involved in photosynthesis were typically the most highly expressed; RuBISCO and chlorophyll a/b binding protein genes were six of the ten most highly expressed genes, exceeding 7,000 RPKM in the case of RuBISCO homolog (LOC_Os12g19381). In roots, three of the top ten most highly expressed genes encoded metallothioneins, cysteine-rich metal chelators associated with heavy metal tolerance [44], and two of these (LOC_Os03g17870, LOC_Os12g38300) were also highly enriched in roots compared to shoots. The most highly expressed gene in rice roots was metallothionein-encoding gene LOC_Os12g38300, which averaged 5,425 RPKM in roots and only 10 RPKM in shoots, a more than 500-fold difference in relative expression. Other genes that were highly expressed in roots but not shoots included several expressed proteins of no known function, a putative aquaporin, several putative protease inhibitors, and a rapid alkalinization protein family member (RALF). Interestingly, RALF protein genes were also among the most highly expressed genes in Arabidopsis pollen [43], and yet the function of RALF proteins remains largely unknown.

To identify cytokinin-responsive genes in rice, we performed differential expression analysis of the RNA-Seq data, analyzing root and shoot samples separately. In roots, the BA treatment caused differential expression (DE) of approximately 4,700 genes (FDR ≤ 0.0001), of which approximately 60% were up-regulated (Additional file 4: Table S4). In shoots, approximately 2,400 genes were differentially expressed, of which 64% were up-regulated (Fig. 1b; Additional file 5: Table S5). There were 609 genes that were DE in both roots and shoots (Fig. 1c), and most of these changed in same direction. However, the magnitude of the change was usually larger in roots than shoots Fig. 1d). The larger number of genes induced in the roots and their greater induction could reflect the mode of application of the hormone: BA was added directly to the hydroponic media bathing the roots and thus the added cytokinin reached the shoots primarily via the transpiration stream.

**Fig. 2:**
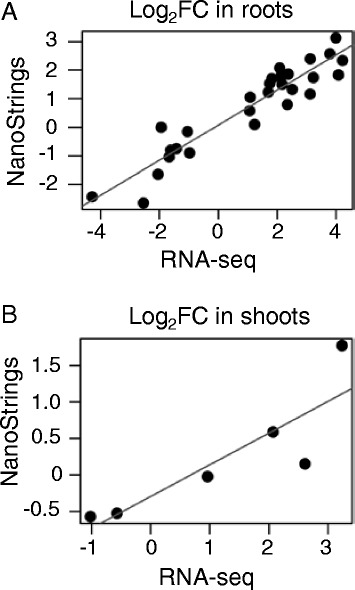
Comparison of gene expression changes detected by NanoStrings and RNA-Seq. Expression of cytokinin-responsive genes in roots (**a**) and shoots (**b**) was assayed using RNA-Seq and Nanostrings on treated samples from two separate experiments. The log_2_ fold change between BA-treated and mock-treated samples was calculated from three biological replicates. Linear regression lines are shown in red. R^2^ was 0.89 and 0.74 for the root and shoot, respectively

To confirm results from the RNA-Seq analysis, we used the NanoString nCounter assay [45, 46] to re-test the response of a subset of genes identified by RNA-Seq as DE in response to cytokinin using three biological replicates distinct from those used for the RNA-Seq analysis. This subset included genes identified as induced or repressed specifically in roots, induced in both tissues, and oppositely regulated in roots and shoots; the subset included genes involved in hormone function (cytokinin, auxin, GA, ethylene, and brassinosteroid), biotic interactions (including five *WRKY* transcription factors), transcription factors, and various other genes (Table 1). Of the 32 genes retested as being DE in roots, 30 were confirmed as DE by cytokinin using the NanoStrings assay (Fig. 2a). Of the eight genes retested as being DE in shoots, six were confirmed using the NanoStrings assay (Fig. 2b). Furthermore, the log_2_ fold changes obtained from NanoStrings and RNA-Seq were similar (Fig. 2). For the comparison of genes identified in roots as DE, the Pearson’s correlation coefficient was 0.93, and for the shoots comparison, the correlation was 0.86. Overall, the NanoStrings assay indicates high confidence in the genes identified as DE in the RNA-Seq dataset. There were 477 genes that were DE in both roots and shoots in the same direction (Additional file 6: Table S6). The list included several genes involved in modulating the response to cytokinin, including eight type-A RRs, four cytokinin oxidases, two cytokinin cytokinin-O-glucosyltransferases, and two genes (*OsHK3* and *OsHK4*) encoding cytokinin receptor histidine kinases (Additional file 6: Table S6 and Additional file 7: Figure S1). The list also included 46 genes annotated as transcription factors [47] from diverse families, with an enrichment for members of the MYB family and two annotated with functions related to ethylene and gibberellin signaling pathways (the gibberellin-associated factor LOC_Os07g39470 was up-regulated; the putative ethylne-responsive transcription factor2 LOC_Os01g54890 was down-regulated). The DE genes also included genes encoding a putative gibberellin receptor, a gibberellin 2-beta-dioxygenase, an ethylene-responsive protein, two ACC oxidase proteins (involved in ethylene biosynthesis) and eight genes annotated with auxin-related functions, including 3 Aux-IAA genes. Two genes annotated as brassinosteroid-insensitive 1-associated receptor kinases were differentially expressed; one was up-regulated and the other down-regulated. Overall, the modulation of expression of these genes suggests that application of cytokinin triggers changes in other hormone-related pathways, highlighting how plant hormone pathways intersect and influence each other. Other notable genes regulated in both roots and shoots included six peroxidases, nine cytochrome P450s, four aquaporins, and two HKT Na^+^ and Na^+^/K^+^ transporters (OsHKT1;5 and OsHKT2;1).

**Table 1:** Log_2_ fold change values for BA-treated samples compared to control samples. Selected genes were retested using NanoString nCounter assay with independent samples, with the same sample number and experimental design used in the RNA-seq experiment. A dash (−) indicates changes that were not significant in a given tissue in the RNA-Seq assay (FDR of 10^−4^). Gene names are shown without “LOC_” prefix for brevity

		Roots		Shoot	
Gene	Description	RNA-seq	Nano	RNA-seq	Nano
Os02g51930	cytokinin-O-glucosyltransferase 2	2.51	1.31	-	-
Os07g13800	cytokinin-N-glucosyltransferase	1.82	1.74	-	-
Os11g12300	NBS-LRR disease resistance protein	2.39	2.01	-	-
Os11g12320	disease resistance protein RPM1	2.14	1.86	-	-
Os03g55080	WRKY3	1.07	0.71	-	-
Os05g46020	WRKY7	3.829	2.73	-	-
Os05g25770	WRKY45	3.24	1.95	-	-
Os09g25070	WRKY62	4.23	2.42	-	-
Os05g09020	WRKY67	3.15	2.50	-	-
Os07g48630	ethylene-insensitive 3	1.09	1.14	-	-
Os02g03410	CAMK_like.12	2.09	2.12	-	-
Os02g53180	1-aminocyclopropane-1-carboxylate oxidase	4.01	3.12	-	-
Os03g29410	tyrosine protein kinase domain containing protein	2.14	1.80	-	-
Os03g42070	Cyclin	−1.94	nd^a^	-	-
Os03g46440	BTBA4 - Bric-a-Brac	2.18	1.56	-	-
Os06g39590	OsIAA23	1.70	1.37	-	-
Os07g11739	cytochrome P450	4.20	1.71	-	-
Os07g40630	BRASSINOSTEROID INSENSITIVE 1 precursor	1.75	1.57	-	-
Os09g38210	auxin efflux carrier component	2.35	1.59	-	-
Os02g13900	BES1/BZR1 homolog protein	1.01	0.80		
Os04g57720	OsRR6	4.89	3.77	3.95	1.21
Os12g43110	OsSAUR58	−1.41	−2.39	-	-
Os10g20260	CSLF7 - cellulose synthase-like family F	−4.29	−3.10	-	-
Os04g44670	AP2 domain containing protein	−2.04	−1.60	-	-
Os04g39980	gibberellin 20 oxidase 2	−1.66	−1.06	-	-
Os04g52670	OsSAUR21	1.02	0.49	0.72	0.68
Os07g48830	glycosyl transferase 8 domain containing protein	3.13	1.14	−0.85	−0.63
Os11g05470	RCN1 Centroradialis-like1 homogous to TFL1	−2.53	−2.75	2.07	0.53
Os01g09640	Myb transcription factor	1.24	0.20	−0.58	−0.56
Os08g42080	ACR5	−0.95	−0.85	3.24	1.85
Os01g55240	gibberellin 2-beta-dioxygenase	−1.04	−0.11	0.97	−0.08
Os07g06860	gibberellin receptor GID1L2	−1.61	−1.62	2.67	ND^b^

^a^none detected in the cytokinin-treated sample

^b^none detected in the either control or cytokinin-treated sample

Most genes that were DE in both roots and shoots changed in the same direction. However, there were 131 genes in which the direction of the change was different in the two tissues (Additional file 8: Table S7). Cytokinin affects root and shoots in different ways [48], and so it was interesting to investigate these genes in detail. Eleven of the 132 genes encoded transcriptional regulators, including the type-B *RR OsRR22* (LOC_Os06g08440), which was down-regulated in roots and up-regulated in shoots. Only one other type-B *RR* (*OsRR26*, LOC_Os01g67770) was regulated by cytokinin, being down-regulated in roots and unaffected in shoots. Other genes that were regulated in opposite directions included ACT domain repeat protein 5 (*ACR5*), likely involved in glutamine-sensing [49], *OsSAUR21*, the phosphatidylethanolamine-binding protein *RCN1*, which is involved in the transition of rice plants to the reproductive phase [50], and several protein kinases and other genes with annotated functions related to signal transduction, including genes involved in calcium signaling.

We examined if the DE genes from rice roots were similar to those differentially regulated by cytokinin in Arabidopsis. To this end, we examined a list of genes robustly regulated by cytokinin in Arabidopsis across several studies, the so-called “golden list” [37] and identified rice counterparts of these genes using orthology annotations from the MSU web site that were generated using OrthoMCL with the default parameters [40, 51]. Approximately half of the Arabidopsis genes on the golden list were annotated with one or more orthologs from rice (116/227). Of these, 55% were differentially expressed in the rice data set in the same direction as in Arabidopsis, and 17% were differentially expressed in opposite directions (Additional file 7: Figure S2). The similarly regulated genes included a number known to negatively regulate cytokinin function, including type-A ARRs and cytokinin oxidases. Thus, a substantial portion of the transcriptional response is conserved between monocots and dicots.

### OsRR22 binds upstream motifs similar to those bound by Arabidopsis type-B RRs

The type-B response regulators are positive elements of cytokinin signaling and are required to modulate transcription of genes in response to the hormone [17, 18, 20, 52]. In Arabidopsis, a common DNA motif sufficient for binding of ARR1, ARR2, ARR10, and ARR11 has been identified as AGAT(T/C), with an extended ARR1 binding motif (AAGAT[T/C]TT) being elucidated due to its enrichment in promoters of known primary response genes [52–55]. Repeats of the extended motif have been incorporated into cytokinin reporters and successfully used to visualize cytokinin activity in plant tissues [56]. More recently, protein binding microarrays have been used to identify transcription factor binding motifs, including those for a subset of the type-B RRs of Arabidopsis (ARR1, ARR2, ARR11, ARR14, ARR18) [57, 58], an approach we chose to use for characterizing type-B RRs of rice.

**Fig. 3:**
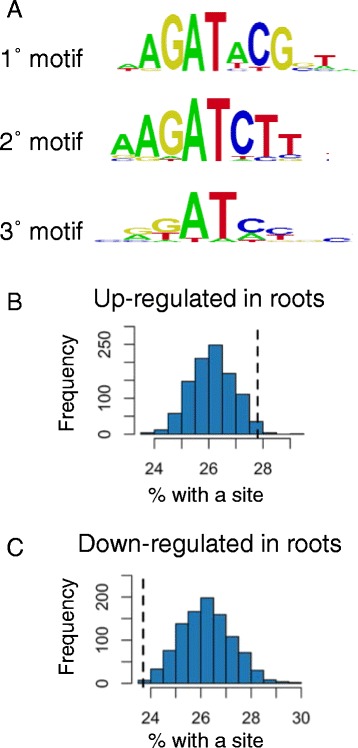
OsRR22 binding sites in differentially expressed genes in rice roots and shoots. **a** Position weight matrix representations of the top-scoring DNA-binding motifs for OsRR22 as determined by the protein binding microarray. **b-c** Percentage of cytokinin-regulated genes in roots that contained one or more of the top 25 8-mer sequences that showed significant binding for OsRR22 (see methods). Percentages indicated with vertical dashed lines were (**b**) 27.8% of up-regulated genes, (**c**) 23.7% of down-regulated genes. Histograms show the distribution of percentages obtained from 1,000 random samples of equivalent size

To determine binding sites of the rice type-B OsRRs, we expressed the DNA-binding domains of OsRR22, OsRR27 and OsRR29 as fusions to maltose-binding protein in vitro and used the protein binding microarray technology (PBM11) to assay binding on all possible 8-mers followed by computational analysis to identify binding motifs [59]. We characterized OsRR22 because it belongs to the same type-B RR subfamily as the Arabidopsis type-B RRs implicated in regulating the cytokinin response, OsRR22 being most similar to AtARR10 and AtARR12. Functional analysis has shown that OsRR22 is able to complement the mutant phenotype of an Arabidopsis *arr1;arr12* loss-of-function line, as well as transactivate *AtARR6-LUC* in transient expression assays, providing evidence of conserved function across monocot and dicot species [27]. Three related binding motifs were identified for OsRR22 (Fig. 3a), two of these with an AGAT core sequence and the third with a palindromic sequence built around the AT core. Significantly, the same motifs have been identified for Arabidopsis type-B RRs of the same subfamily [57], indicating a conservation of binding between the rice and Arabidopsis type-B RRs implicated in regulating the cytokinin response. In contrast to OsRR22, OsRR27 and OsRR29 belong to monocot-specific subfamilies of type-B RRs and contain substantially diverged Myb-like DNA-binding domains [27]. No significantly enriched motifs were identified for OsRR27 and OsRR29, although both produced fusion proteins at similar levels to those found with OsRR22 (Additional file 7: Figure S3). OsRR27 and OsRR29 may require additional sequences outside the Myb-like domain, additional cofactors and/or plant-specific post-translational modifications to allow for binding to DNA.

We searched for instances of the OsRR22 binding motifs within the putative regulatory regions (1 kb upstream of the transcriptional start site) of cytokinin-regulated genes, the prediction being that direct targets of the type-B RRs would be enriched for the binding motifs. Of the 2,890 genes that were up-regulated in roots, 27.8% contained one or more of the OsRR22-like extended binding motifs. To determine if this percentage was unusually large or small, we generated 1,000 random samples of 2,890 rice genes and for each sample, calculated the percentage of genes that contained an OsRR22 binding site. Out of 1,000 random samples of 2,890 genes, only 2.2% contained a higher percentage of genes with OsRR22 binding sites than the set of 2,890 genes that were up-regulated in roots (Fig. 3b). By contrast, of the 1,780 genes that were down-regulated in roots, only 23.7% contained an OsRR22 binding site (Additional file 7: Fig. 3c). To assess the significance of this, we repeated the random sampling experiment, selecting 1,000 random samples of 1,780 genes and for each sample, calculating the percentage of genes with one or more OsRR22 sites. Of the 1,000 random samples, only 1% contained a lower percentage of genes with an OsRR22 binding site than the set of 1,780 genes that were down-regulated by cytokinin. We observed similar, but less significant trends for differentially expressed genes in shoots. Thus, OsRR22 binding sites were unusually prevalent among up-regulated but not down-regulated genes, consistent with OsRR22 being primarily a positive regulator of cytokinin signaling. However, many DE genes (more than 70%) lacked an extended OsRR22 binding motif. These genes may contain only the core type-B RR binding motif (which, although sufficient for binding, is too short to be diagnostic for enrichment), a type-B RR binding site diverged from this consensus, a type-B RR binding site outside of the 1-kb window examined, or may be dependent on other factors besides the type-B RRs for regulation by cytokinin.

### Gene Ontology enrichment analysis of cytokinin-regulated genes in rice

To explore the biological processes in which cytokinin participates in rice, we conducted a Gene Ontology enrichment analysis on genes DE in response to cytokinin. We first examined genes induced or repressed by cytokinin in both root and shoot tissues (Additional file 7: Figures S4 and S5). Among the induced genes, as expected, we saw an enrichment of genes involved in two-component signaling and signal transduction (Additional file 7: Figures S1 and S4). Additionally, we observed a significant enrichment in genes involved in adenine and purine salvage, processes that have been implicated in the interconversion of cytokinin (CK) bases, ribosides, and nucleotides [60]. Similar to GO term enrichment studies in Arabidopsis treated tissues [34, 37, 38, 61], genes involved in secondary metabolic processes were enriched among the genes induced in both roots and shoots. Among the genes repressed in both roots and shoots, the most significant enrichment was for GO terms related to the response to oxidative stress (Additional file 7: Figure S5).

We next examined GO term enrichment among the genes DE in the root tissue. Consistent with cytokinin regulating a transcriptional cascade, there is an enrichment of genes with a role in the regulation of transcription among the genes induced by cytokinin in the root (Additional file 7: Figure S6), with 550 genes encoding transcription factors showing DE in response to cytokinin out of a total of 1,611 transcription factors encoded by the rice genome [62]. Thus, remarkably, more than one-third of rice transcription factors are differentially expressed in response to cytokinin in rice roots. This suggests large scale changes in the rice root transcriptome following cytokinin treatment, consistent with the large number of genes we observe as differentially expressed in response to cytokinin.

There is a strong enrichment for genes involved in post-translational modification of proteins among the genes induced by cytokinin in the root, specifically in ubiquitination and phosphorylation (Additional file 7: Figure S6), the former perhaps indicating a role for cytokinin in regulating protein turnover in rice. Several studies have linked cytokinin to the regulation of protein turnover in Arabidopsis, although such a strong GO enrichment as we find in rice has not been previously noted in other cytokinin transcriptome studies. In Arabidopsis, the RPN12 subunit of the 26S proteasome plays a role in regulating cytokinin sensitivity [63]. In addition, the cytokinin signaling pathway itself is subject to regulation by protein turnover. The turnover of a subset of type-A ARR proteins occurs through a Rub protein modification-dependent pathway [64] and is regulated by cytokinin via differential phosphorylation of the conserved Asp residue [21]. Cytokinin transiently stabilizes degradation of ARR1 in Arabidopsis [65], a type-B RR, whose turnover is also regulated by the KISS ME DEADLY family of F-box-proteins [66]. In contrast, cytokinin is proposed to promote the 26S proteasome-dependent turnover of ARR2 via regulation of phosphorylation of the conserved Asp target [67]. Cytokinin also regulates the turnover of other proteins. For example, cytokinin regulates the turnover of ACC synthase, which catalyzes the key step involved in ethylene biosynthesis [68, 69]. Further, during lateral root development in Arabidopsis, cytokinin targets dephosphorylated PINs present preferentially on anticlinal membranes for lytic degradation [70]. Together, these studies from Arabidopsis indicate that cytokinin regulates the turnover of its own signaling pathway, as well as additional downstream targets. The large number of genes involved in protein degradation regulated by cytokinin in rice suggests that the modulation of protein turnover may be a prominent mechanism by which cytokinin regulates cell function in rice.

One intriguing category of genes enriched among those induced by cytokinin in the root encode proteins involved in the synthesis of siderophores (Additional file 7: Figure S6). Siderophores are small, high-affinity iron chelating compounds secreted by graminaceous plants to increase the uptake of these minerals [71–73]. In rice these compounds are secreted at lower levels than in other grasses and may be more involved in the uptake of zinc rather than iron [74]. While cytokinin has been linked to the uptake of other minerals, including nitrate, phosphate, sulfur, and iron [4], it has not previously been linked to zinc uptake.

Finally, we examined GO enrichment among the genes DE by cytokinin in the shoot. In addition to the previously mentioned two-component signaling and adenine and purine salvage, we observed an enrichment of genes induced by cytokinin that are involved in the regulation of cell growth, lipid metabolism, and secondary metabolism (Additional file 7: Figure S7). The GO term secondary metabolic processes is also enriched in cytokinin-treated Arabidopsis tissues [37, 38, 61]. Genes involved in the synthesis of siderophores are enriched among the down-regulated genes (Additional file 7: Figure S8), which is distinct from the induction of these genes by cytokinin in roots (see above). Other enriched terms include flower development and protein phosphorylation.

**Fig. 4:**
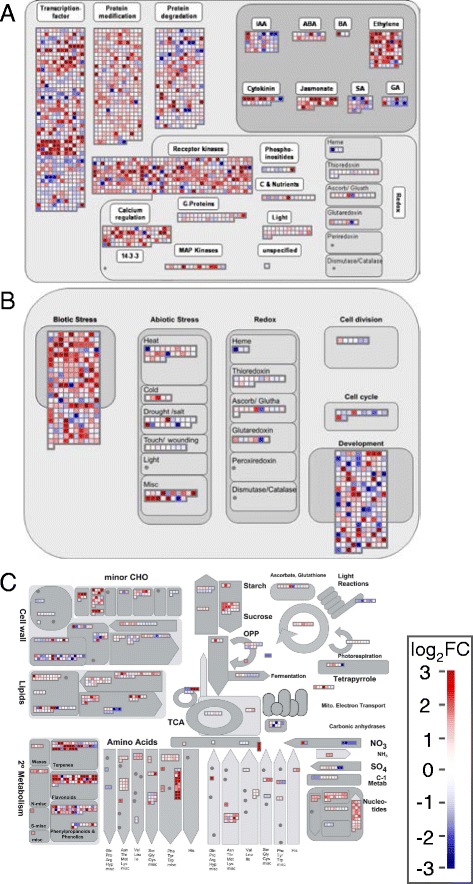
Mapman categorization of genes differentially regulated by cytokinin. MapMan representations of overview of regulation (**a**), cellular response (**b**) and metabolism (**c**) of genes identified as differentially regulated in response to cytokinin in rice or roots. Differentially expressed genes are shown as color-coded squares. *Blue* indicates down-regulated genes and *red* indicates up-regulated genes with the relative level of log_2_ fold change indicated by the scale shown

To further investigate the functions of cytokinin-regulated genes, we analyzed differentially expressed genes in rice roots (Fig. 4a-c) and shoots (Additional file 7: Figure S9) using functional annotations from MapMan [75]. In general, the enrichment analysis using MapMan categories correlated well with results observed in GO analysis. As expected, there were a large number of genes linked to development differentially regulated by cytokinin in both roots and shoots (Figs. 4a and Additional file 7: Figure S8). Many genes involved in hormone signaling and metabolism were regulated in both the root and shoot, most markedly those related to ethylene (Figs. 4a and Additional file 7: Figure S8), the majority of which were induced by cytokinin. There was also a consistent up-regulation of genes involved in jasmonate function, and general down-regulation of genes involved in gibberellin function. Cytokinin also affected many genes involved in auxin, ABA, and salicylic acid function in the root.

**Fig. 5:**
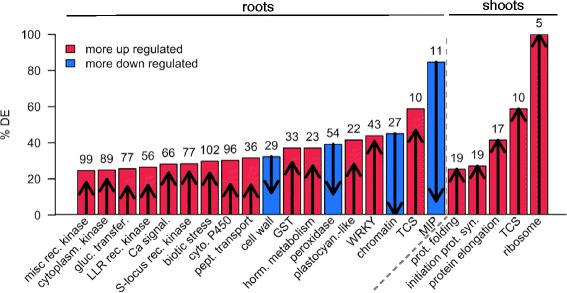
MapMan categories enriched in cytokinin-responsive genes in rice tissues. Representation of the MapMan categories enriched in the number of cytokinin-regulated genes in rice roots (*left*) and shoots (*right*). The numbers (above each bar indicate the number of cytokinin-regulated genes per category. Arrow length and direction and bar shading indicate how many differential expressed genes were up-regulated (*red bar, arrows pointing up*) or down-regulated (*blue bar, arrows pointing down*). Categories are ordered by significance (FDR) with the most significant terms on the left. Only categories with FDR 0.001 or smaller are shown

As observed with the GO enrichment analysis described above, there were a large number of genes involved in the regulation of transcription and genes linked to protein degradation regulated by cytokinin in rice roots (Fig. 4a). Interestingly, many kinases, especially receptor-like kinases, were up-regulated in roots, which is consistent with the over-representation of the GO term protein phosphorylation noted above and which suggests that cytokinin may regulate cellular function through modification of Ser/Thr phosphorylation of many downstream targets. Consistent with a role as a negative regulator of cell proliferation in roots, multiple genes involved in cell division and the cell cycle were down-regulated by cytokinin (Fig. 4b). MapMan analysis also identified an unusually large percentage of cytokinin-regulated genes related to stress-response functions (Figs. 4b and 5). These genes included those involved in the response to biotic stimuli and abiotic stimuli (heat, cold, and drought/salt) with the majority found to be up-regulated by cytokinin. There was also an enrichment of genes involved in calcium and G-protein signaling (Fig. 4a), suggesting that cytokinin may crosstalk with these key signaling pathways. Other enriched categories of genes included, peroxidases and GSTs, and genes involved in cell wall function (Fig. 4c).

Using statistical analysis of MapMan annotations, we identified MapMan categories that contained an unusually large number of differentially expressed genes (Fig. 5 and Additional file 9: Tables S8 and Additional file 10: Table S9). Members of the WRKY transcription factor family contained an unusually large number of DE genes in roots (Fig. 5), consistent with a modulation of a genes involved in biotic stress response. Several categories of kinases were overrepresented, including LRKs, S-locus receptor kinases. Other notable categories of genes enriched in cytokinin-induced genes in roots were cytochrome P450s and genes involved in calcium signaling. Among the down-regulated genes in the roots, genes involved in cell wall function, chromatin modification, and peroxidases were enriched. In the shoots, genes involved in protein function (folding, initiation of protein synthesis and elongation) and the ribosome were overrepresented.

### Cytokinin-regulated genes overlap with genes driving defense responses

**Fig. 6:**
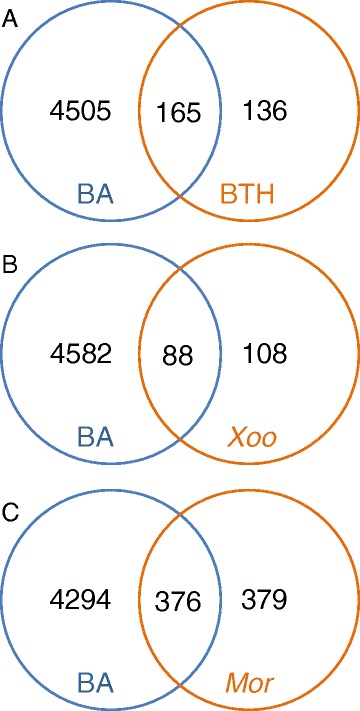
Similarity between biotic stress-regulated and cytokinin-regulated gene. expression in rice. Overlap of the differentially expressed genes in response to benzyladenine (BA) in roots and (**a**) benzothiadiazole (BTH) [76], (**b**) *Xanthomonas oryzae* pv. oryzicola (*Xoc*) [77] or (**c**) *Magnaporthe oryzae* (*Mor*) [78]. Previously reported biotic stress data are from microarray experiments on whole seedlings. Only genes that could be assayed by a single, non-cross hybridizing probe on the array are shown

Our analysis suggests a substantial overlap in the genes regulated by cytokinin in rice and those involved in biotic stress responses. To further explore this, we examined publicly available expression data related to pathogen interactions in rice for overlap with the cytokinin-related gene changes found here by RNA-Seq. We first examined gene expression changes in response to benzothiadiazole (BTH) [76], which activates the salicylic acid pathway, mimicking a plant’s natural defense response. More than half of BTH-regulated genes were also regulated by cytokinin in roots (Fig. 6a), suggesting that cytokinin activates a substantial portion of the BTH targets in roots. We also examined the overlap of the cytokinin-regulated transcriptome in roots with changes in the transcriptome in response to the plant pathogens *Xanthomonas oryzae* pv. *oryzicola* (*Xoo*) [77] and *Magnaporthe oryzae* [78], the causative agents of rice blight and rice blast respectively. Similar to the BTH results, nearly half of genes affected by the bacterial pathogen *Xoo* or by the fungal pathogen *M. oryzae* were also differentially expressed in the cytokinin RNA-Seq experiment (Fig. 6b-c). This indicates that cytokinin signaling alters a major fraction of the pathogen-regulated transcriptome, suggesting that cytokinin plays a crucial role in the regulation of genes driving the response to biotic stress.

Infection by *M. oryzae* has recently been shown to elevate iP-type cytokinins in rice leaf blades [79]; altered cytokinin levels have been observed in many other plant pathogen interactions [4]. Further, *M. oryzae* itself has been shown to synthesize various cytokinin species [79]. As was found in Arabidopsis [80], cytokinin acts synergistically with salicylic acid to activate defense responses in rice, in a manner dependent on both *WRKY45* and *OsNPR1* [79]. The authors suggest that, similar to the interaction of Arabidopsis with the pathogen *Hyaloperonospora arabidopsidis* [80], *M. oryzae* likely elevates cytokinin in order to increase its proliferation by modification of host processes such as increasing sink activity; rice perceives this inappropriate modulation of cytokinin levels and activates defense responses [79].

Many resistance genes in plants encode nucleotide-binding site leucine-rich repeat (NBS-LRR) proteins, which are involved in the detection of diverse pathogens, including bacteria, viruses, fungi, nematodes, insects and oomycetes [81]. Consistent with a link between cytokinin and pathogen response signaling, many NBS-LRR genes were regulated by cytokinin in rice, including 26 genes related to the Arabidopsis *RPM1/*(*RPS3*) gene and several other *RPS* homologs. Further, the induction of a large fraction of the receptor-like kinases (RLKs) in rice by cytokinin (Fig. 4a) may reflect the intersection with pathogen signaling as it has been postulated that the expansion of the rice RLK gene family after the rice/Arabidopsis split involves defense-related genes [82].

**Fig. 7:**
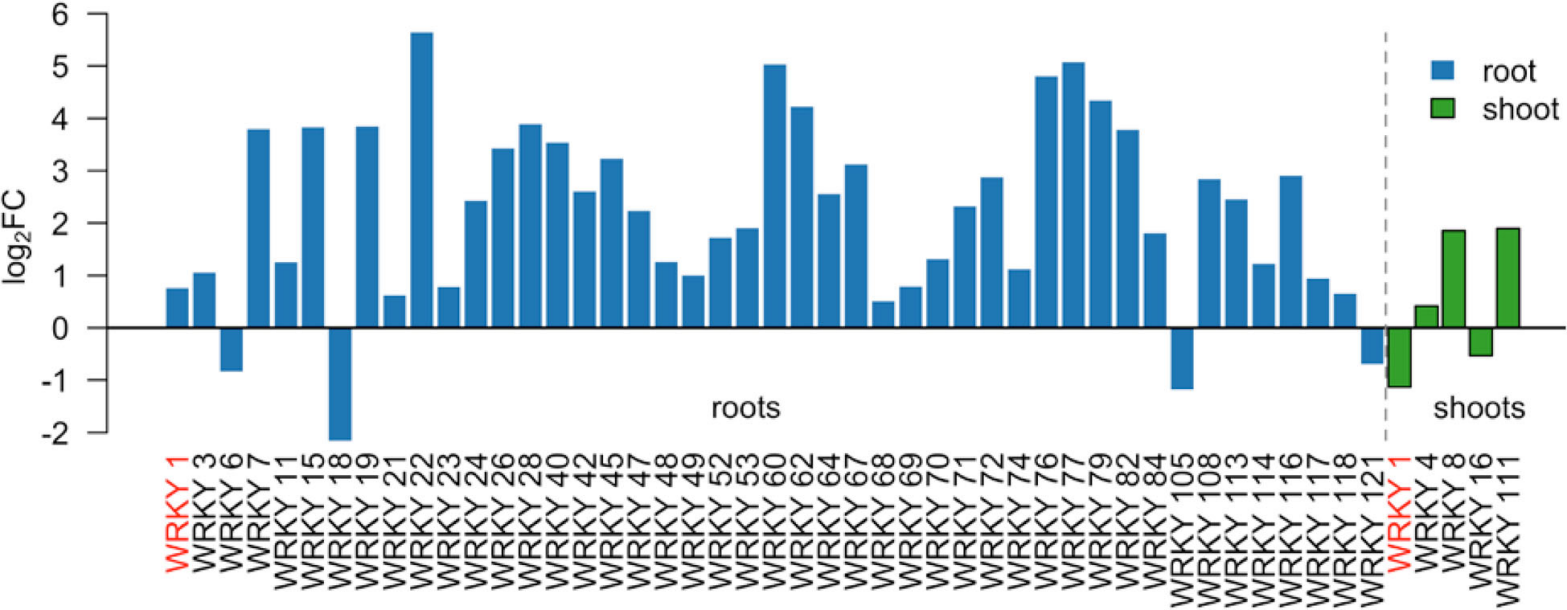
Cytokinin regulation of WRKY transcription factors in rice. Log_2_ fold change in the level of the *WRKY* transcription factors that are differentially expressed in rice roots (*blue bars*) or shoots (*green bars*) in response to cytokinin treatment. WRKY1 (*indicated in red*) is regulated in both tissues

The WRKY family has been closely linked to biotic stress response. Many rice WRKYs are rapidly regulated transcriptionally upon pathogen infection [78, 83] and many have been shown to directly affect susceptibility to pathogens [84]. In our RNA-Seq experiment, a large number of *OsWRKYs* were up-regulated by cytokinin, particularly in the root tissue (Fig. 7). The rice genome contains 98 to 102 *OsWRKY* transcription factors [85] and almost half (50/102) were differentially expressed in BA-treated rice roots, and most of these (45/50) were up-regulated. In rice, multiple *WRKY* loci have been linked to pathogen responses [86], and several of these are induced by cytokinin, including *OsWRKY28*, *OsWRKY45, OsWRKY45, OsWRKY53, OsWRKY62*, *OsWRKY71*, *OsWRKY76* (Fig. 7). For example, overexpression of *OsWRKY53* and *OsWRKY71* increased resistance to the fungal and bacterial pathogens, respectively [87, 88]. Overexpression of *OsWRKY28*, *OsWRKY62*, *OsWRKY71*, and *OsWRKY76* resulted in increased resistance to *Xoo* [89]. The *WRKY45* gene, which is induced 9.5-fold in response to cytokinin treatment in rice roots (Fig. 7), plays key role in the response to both *Xoo* and *M. oryzae*: overexpression of *WRKY45* conferred strong resistance to the Xoo and *M. oryzae* [90]; *OsWRKY45* is essential for BTH-primed plant immunity to *M. oryzae* and *Xoo* [91]. WRKY45-2, WRKY13, and WRKY42 form a sequential transcriptional regulatory cascade required for resistance to *M. oryzae* [86]. Further, the role of cytokinin in defense responses has been linked to *WRKY45* as cytokinin and salicylic acid act synergistically to elevate genes encoding the enzymes involved in the biosynthesis of diterpenoid phytoalexin defense compounds in a *WRKY45*-dependent manner [92]. Together, these data suggest that cytokinin plays an important role in facilitating the response to pathogen in rice, particularly by the regulation of the NBS-LRR receptors and the WRKY family of transcription factors. Analysis of rice lines disrupted for cytokinin metabolism and signaling should help elucidate the complex interactions among cytokinin, defense signaling and the NBS-LRR/WRKYs.

## Conclusions

Here we demonstrate that there are differences in effects of cytokinin on the transcriptome of rice and Arabidopsis, including both a large increase in the number of genes induced by cytokinin in rice and differences in the suites of genes regulated, which suggests distinct cytokinin outputs in monocots. One of the most enriched sets of genes regulated by cytokinin in rice are linked to biotic responses, and there is substantial overlap between genes regulated by cytokinin and by various pathogens, suggesting a close link between cytokinin and pathogen responses in rice. These results begin to unravel the complex gene regulation after cytokinin perception in a crop of agricultural importance and provide insight into the processes and responses modulated by cytokinin in monocots.

## Methods

### Rice RNA-Seq and NanoString analysis

Rice seeds (Nipponbare) obtained from plants grown in our research greenhouse were surface sterilized with 2.5% sodium hypochlorite for 15 min and germinated on filter paper containing distilled water at 37 °C for one day. Germinated seeds were transferred to hydroponic culture containing Kimura B solution [93] in a growth chamber set to 14 h light (28 °C) and 8 h of dark (23 °C) with light intensity 700 μmol m^−2^ s^−1^. Plants were grown in two pots. At day 10 of hydroponic cultivation, culture media was replaced with new media containing 5 μM BA or 0.05 mN NaOH as a control. Following 120 min of the treatment, roots and shoots were harvested separately. Libraries were made from each of the four rice sample types (treated root, treated shoot, untreated root, and untreated shoot) using the ThruPLEX DNA-seq kit (Rubicon Genomics). The multiplexed samples were combined and sequenced on three lanes of an Illumina HiSeq flowcell for 100 cycles, yielding 100 base, single end reads. Sequencing was done at the David H. Murdock Research Institute in Kannapolis. Each lane contained one library from each of the four sample types.

The NanoString nCounter analysis was performed by the University of North Carolina Genomics and Bioinformatics Core Facility. 160 ng of total RNA was directly hybridized with gene specific color-coded probes and the data collection is carried out in the nCounter Digital Analyzer as described by the manufacturer (NanoStrings Technologies; Seattle, WA). The NanoString Codeset (Additional file 11: Table S10) was designed and synthesized by NanoString Technologies (Seattle, WA), which including three reference genes, LOC_Os01g16414 (Actin), LOC_Os02g32030 (Elongation factor), and LOC_Os05g01600 (Actin7). In addition, six positive-control and eight negative-control probes were added to each reaction to produce a standard curve for normalization. All the reaction counts were within the linear dynamic range of the standard curve. For each gene analyzed, the average plus two times standard deviation of the negative controls was subtracted from the raw data and then normalized to the standard curve within each reaction and three reference genes.

### Sequence processing

Sequences from rice were aligned onto the *O. sativa* japonica genome assembly Os-Nipponbar-Reference-IRGSP-1.0 (released October 2011) [40] using tophat (v.2.0.5) [94] and bowtie [94]. For the tophat alignment, the default parameters were used except that the maximum intron size was set to 5000. The default settings used allowed up to two mismatches per read. The analysis was limited to reads with exactly one reported alignment, using the SAM format NH tag to distinguish such reads. Alignment, splice junction, and coverage graph files were deployed on an IGB QuickLoad site to facilitate visualization in Integrated Genome Browser [39]. The number of single-mapping reads that overlapped each annotated protein-coding gene in the MSU Rice Genome Annotation Project (Release 7) genome annotations (RGAP7) was calculated using the samtools view –c function and supplied as inputs for differential expression analysis.

### Differential expression analysis

Differentially expressed genes were identified using edgeR generalized linear modeling and exactTest methods. Differential expression was examined in roots and shoots (separately) between treated and untreated samples. In all cases, only genes with at least one read in one of the compared samples were considered (i.e. if a gene had no reads at all in any sample being compared, we did not test it for differential expression). An FDR cutoff of 0.0001 was used to determine differential expression of individual genes. Code used to process and analyze data is available from https://bitbucket.org/lorainelab/ricecyto.

### Determination of rice Type-B DNA binding motifs

The DNA binding domains for the three rice type-B RRs (RR22, RR27, RR29) were fused to MBP by cloning into pMAL-c2x using restriction enzymes BamH I and Sal I. Primers used for cloning were as follows: CCCGGATCCTCAGCTGCAAAGAAGCCC and GTCGACTCAACCTAGTCTCTTGAGGTAAAG for RR22; CCCGGATCCAGGTTCACATGGACGACG and GTCGACTCATCTGTATTTCTGTAGATGGCT for RR27; CCCGGATCCACTAAGAAGAAATATTATCTCATG and GTCGACTCACAAATCCTTTGTTAGCCGTAG for RR29. Constructs were verified by sequencing and plasmids were introduced into the BL-21 strain of *E. coli* for protein expression. Expression of recombinant proteins was performed using the Zymo Dual Media Kit (Zymo Research). Induction was carried out using 0.1 mM IPTG for 8 h at 37 °C. Total soluble protein extracts from *E. coli* cultures expressing the recombinant proteins were analyzed by gel electrophoresis and immunoblot to confirm induction efficiency. OsRR22, OsRR27 and OsRR29 DNA binding specificities were then determined using protein binding microarrays (PBM11) as described by [59]. The complete list of 25 8-mers and their E-scores can be found in the project repository PbaAnalysis/data/OsRR22_results.txt under PbaAnalysis/data/OsRR22_results.txt.

### Gene Ontology and MapMan gene category enrichment analysis

Gene families with unusually many or few differentially expressed genes were detected using GOSeq, which accounts the tendency of genes with larger transcripts to be preferentially detected as differentially expressed in RNA-Seq data. GO term trees were visualized using Virtual Plant. MapMan gene to category assignments for MSU7 rice annotations were downloaded from the MapMan Web site at http://mapman.gabipd.org/ as plain text files. Gene Ontology annotations were obtained from the Gene Ontology Web site. Categories containing unusually many differentially expressed genes were identified using GOSeq; the code is available in the project repository PbaAnalysis/data/OsRR22_results.txt. The MapMan desktop application was used to visualize.

## Additional files

Additional file 1: Table S1. Number of rice RNA-Seq reads mapping to genes by library. (XLSX 12 kb)
Additional file 2: Table S2. Rice RNA-Seq gene expression values in RPKM. (XLSX 13 kb)
Additional file 3: Table S3. Genes that were DE between roots and shoots in rice. (XLSX 3612 kb)
Additional file 4: Table S4. Genes differentially regulated in response to cytokinin in roots. (XLSX 10749 kb)
Additional file 5: Table S5. Genes differentially regulated in response to cytokinin in shoots. (XLSX 1485 kb)
Additional file 6: Table S6. Genes that were DE in roots and shoots in the same direction. (XLSX 419 kb)
Additional file 7: Figure S1. Regulation of genes involved in cytokinin biosynthesis, signaling, response, and degradation. **Figure S2.** Cytokinin regulation of orthologous genes in rice and Arabidopsis. **Figure S3.** Characterization of rice type-B RRs OsRR27 and OsRR29 through use of protein binding microarrays. **Figure S4.** GO categorization of genes induced by cytokinin in both the root and the shoot. **Figure S5.** GO categorization of genes repressed by cytokinin in both the root and the shoot. **Figure S6.** GO categorization of genes induced by cytokinin in the root. **Figure S7.** GO categorization of genes induced by cytokinin in the shoot. **Figure S8.** GO categorization of genes repressed by cytokinin in the shoot. **Figure S9.** MapMan overviews of metabolism, cellular response and regulation of cytokinin-regulated genes in shoots in rice. (XLSX 220 kb)
Additional file 8: Table S7. Genes that were DE in roots and shoots in opposite directions. (XLSX 58 kb)
Additional file 9: Table S8. MapMan categories and DE genes from roots. (PDF 1088 kb)
Additional file 10: Table S9. MapMan categories and DE genes from shoots. (XLSX 26 kb)
Additional file 11: Table S10. Primers used in the NanoString analysis. (XLSX 44 kb)

## Abbreviations

BA: Benzyladenine
DE: Differential expression
GA: Gibberellic acid
HKs: Histidine kinases
HPs: Histidine phosphotransfer proteins
IGB: Integrated Genome Browser
RPKM: Reads per kilobase of expressed sequence per million mapped reads
RRs: Response Regulators
