# Supplementary material for: Characterization of the cytokinin-responsive transcriptome in rice
Source: BMC Plant Biol. 2016 Dec 8;16:260. doi: 10.1186/s12870-016-0932-z (PMC5146874; doi:10.1186/s12870-016-0932-z)
Supplement: Additional file 9: Table S8. — MapMan categories and DE genes from roots. (PDF 1088 kb) [file 12870_2016_932_MOESM7_ESM.pdf]

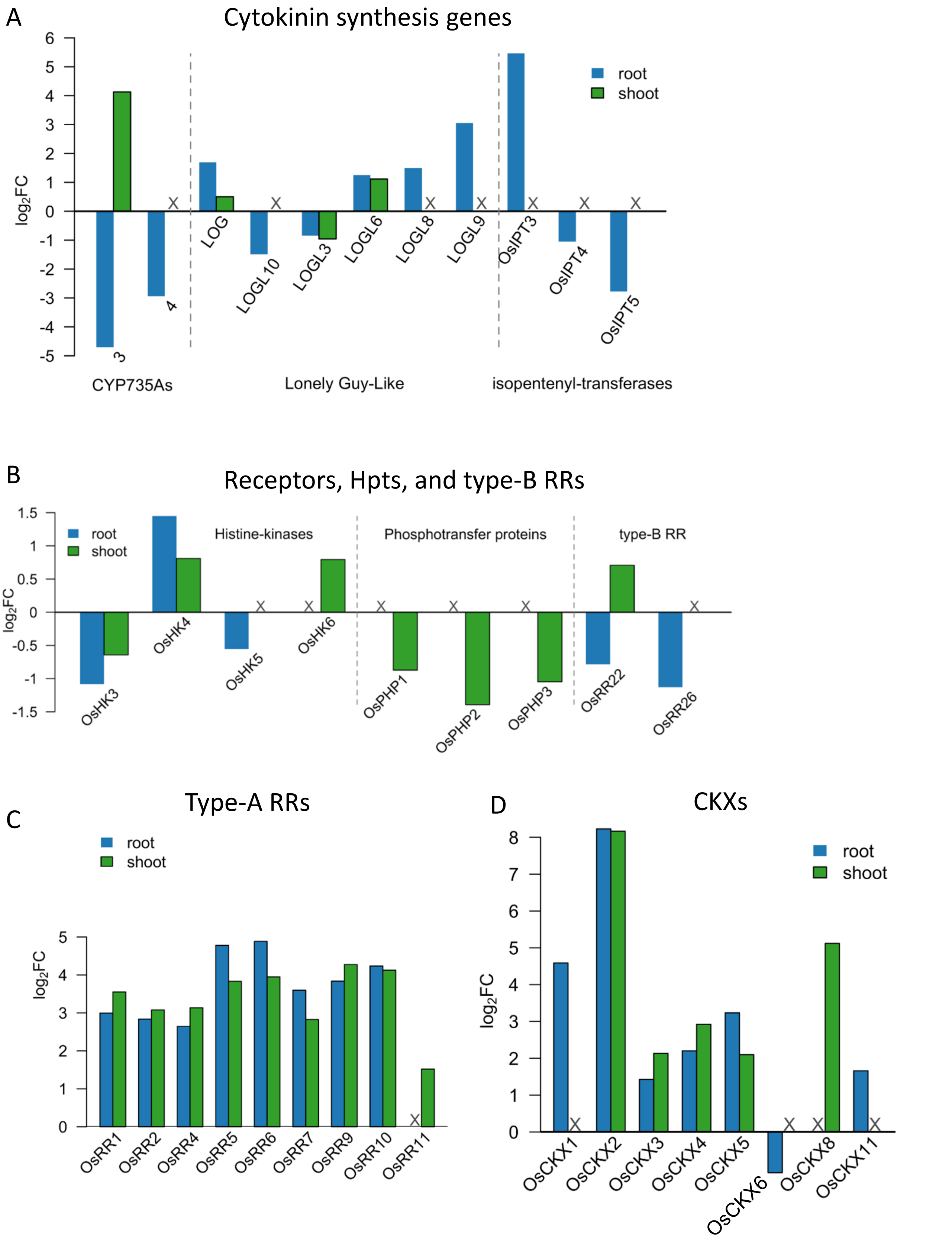

Figure S1. Regulation of genes involved in cytokinin (A) biosynthesis, (B) signaling, (C) response, and (D) degradation. Log2-fold change in the BA-treated samples relative to the mock control samples are indicated. Only genes that are differentially expressed between the control and cytokinin-treated samples are shown. Green boxes indicate genes differentially regulated in shoots; blue boxes in roots.

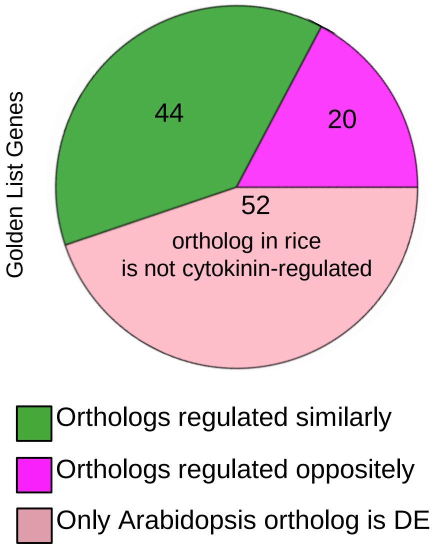

Figure S2. Cytokinin regulation of orthologous genes in rice and Arabidopsis. Comparison of the regulation of the rice orthologs of the robustly cytokinin-regulated genes (aka the "golden list") in Arabidopsis discovered in a meta-analysis of Arabidopsis transcriptome studies [36].

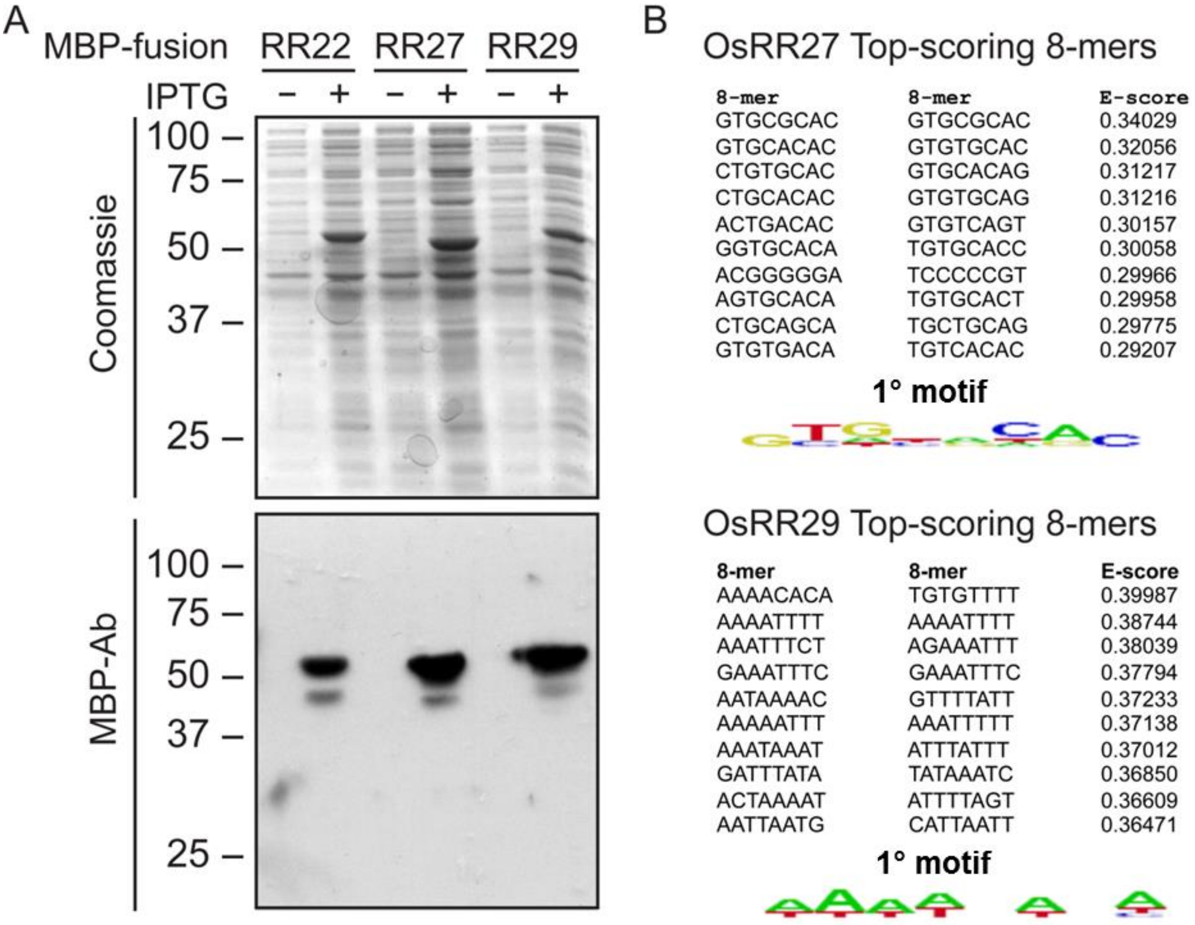

Figure S3. Characterization of rice type-B RRs through use of protein binding microarrays. (A) Expression of the DNA-binding domains for RR22, RR27, and RR29 as fusions to maltose binding protein (MBP) in *E. coli*. A Coomassie Blue-stained gel of total bacterial protein is shown before (-) and after (+) IPTG induction of the fusion proteins (top panel) as well as immunoblot analysis for the fusion proteins with an anti-MBP antibody (lower panel). The fusion proteins are predicted to have molecular masses of 50.7 kDa (MBP-RR22), 49.4 kDa (MBP-RR27), and 50.5 kDA (MBP-RR29). (B) OsRR27 and OsRR29 do not display preferential binding to any particular DNA motif.

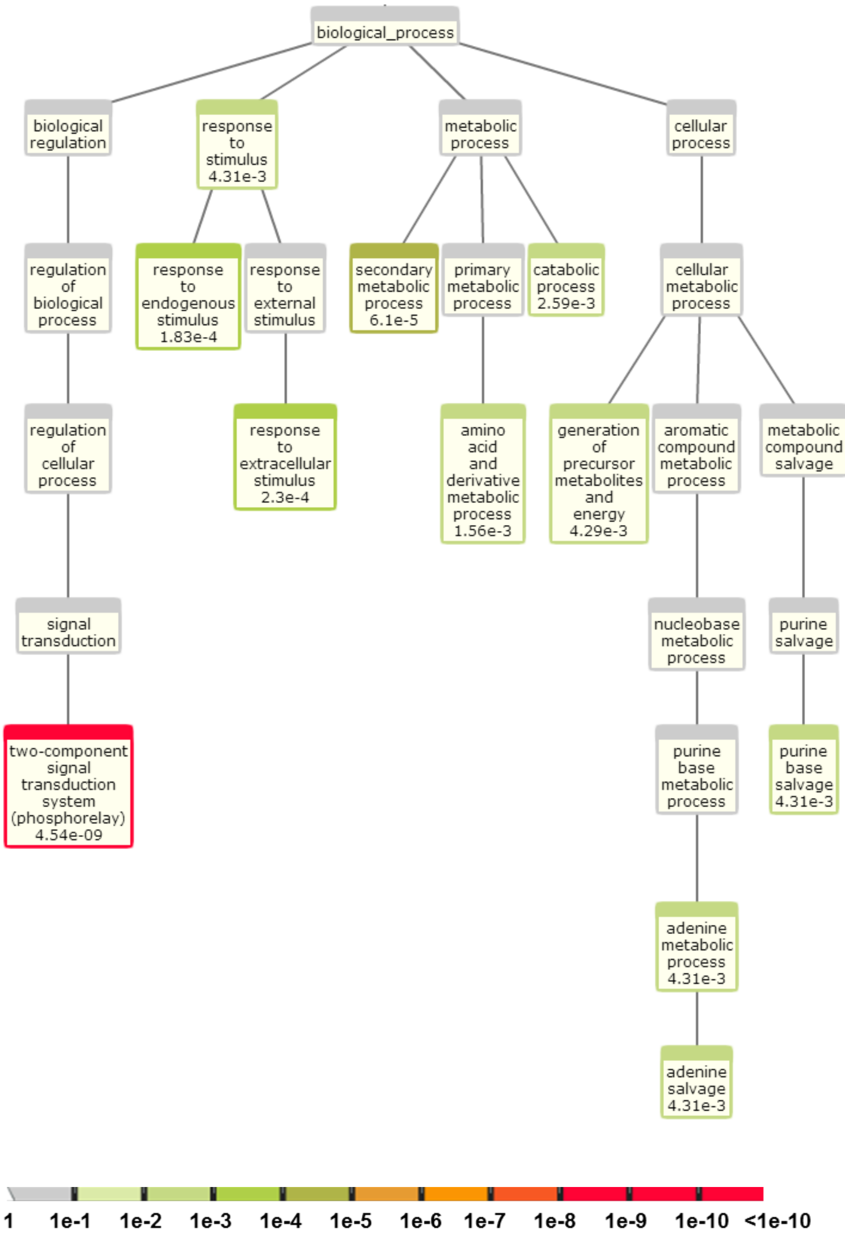

Figure S4: GO categorization of genes induced by cytokinin in both the root and shoot Using the VirtualPlant (http://virtualplant.bio.nyu.edu) tool for gene ontology (GO) term enrichment, analysis was performed on genes induced by cytokinin in both the root and shoot tissues of rice. All terms have p-value cutoff of < 0.01 and the p-value of the GO group is depicted by the color scale below.

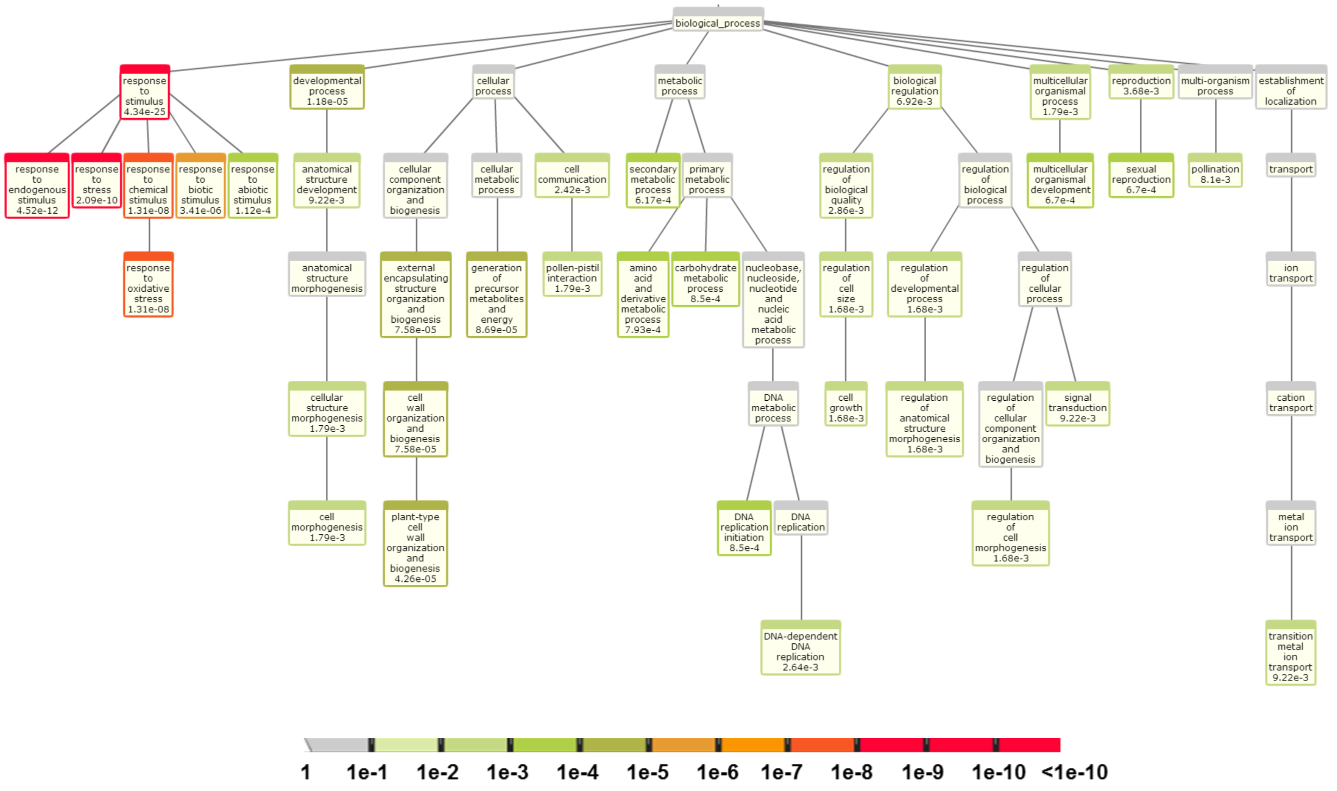

Figure S5: GO categorization of genes repressed by cytokinin in both the root and shoot Using the VirtualPlant (http://virtualplant.bio.nyu.edu) tool for gene ontology (GO) term enrichment, analysis was performed on genes repressed by cytokinin in both the root and shoot tissues of rice. All terms have p-value cutoff of < 0.01 and the p-value of the GO group is depicted by the color scale below.

A

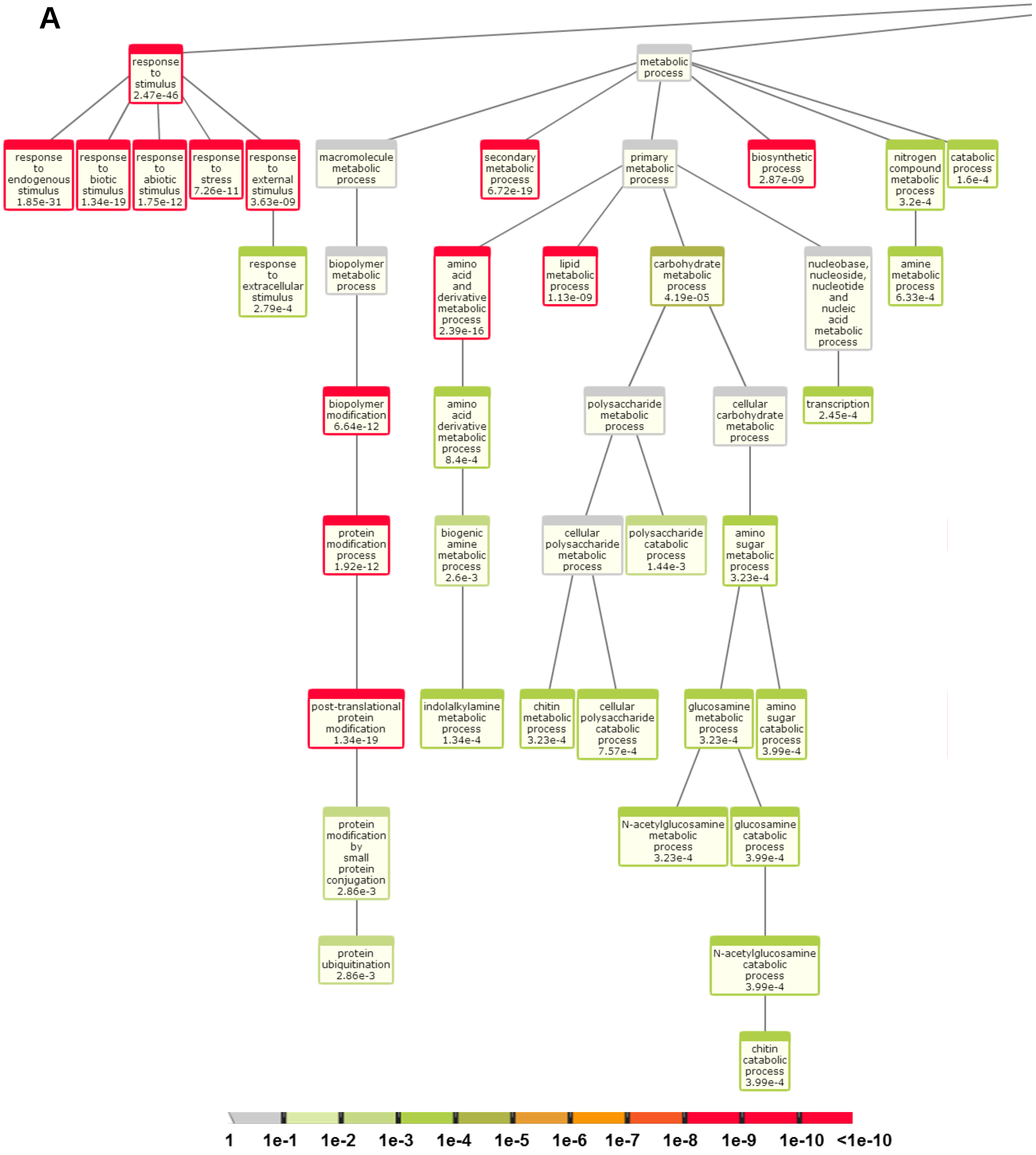

response to stimulus 2.47e-46
metabolic process
response to endogenous stimulus 1.85e-31
response to biotic stimulus 1.34e-19
response to abiotic stimulus 1.75e-12
response to stress 7.26e-11
response to external stimulus 3.63e-09
macromolecule metabolic process
secondary metabolic process 6.72e-19
primary metabolic process
biosynthetic process 2.87e-09
nitrogen compound metabolic process 3.2e-4
catabolic process 1.6e-4
response to extracellular stimulus 2.79e-4
biopolymer metabolic process
amino acid and derivative metabolic process 2.39e-16
lipid metabolic process 1.13e-09
carbohydrate metabolic process 4.19e-05
nucleobase, nucleoside, nucleotide and nucleic acid metabolic process
amine metabolic process 6.33e-4
biopolymer modification 6.64e-12
amino acid derivative metabolic process 8.4e-4
polysaccharide metabolic process
cellular carbohydrate metabolic process
transcription 2.45e-4
protein modification process 1.92e-12
biogenic amine metabolic process 2.6e-3
cellular polysaccharide metabolic process
polysaccharide catabolic process 1.44e-3
amino sugar metabolic process 3.23e-4
post-translational protein modification 1.34e-19
indolalkylamine metabolic process 1.34e-4
chitin metabolic process 3.23e-4
cellular polysaccharide catabolic process 7.57e-4
glucosamine metabolic process 3.23e-4
amino sugar catabolic process 3.99e-4
protein modification by small protein conjugation 2.86e-3
N-acetylglucosamine metabolic process 3.23e-4
glucosamine catabolic process 3.99e-4
protein ubiquitination 2.86e-3
N-acetylglucosamine catabolic process 3.99e-4
chitin catabolic process 3.99e-4
1
1e-1
1e-2
1e-3
1e-4
1e-5
1e-6
1e-7
1e-8
1e-9
1e-10
<1e-10

Figure S6a

B

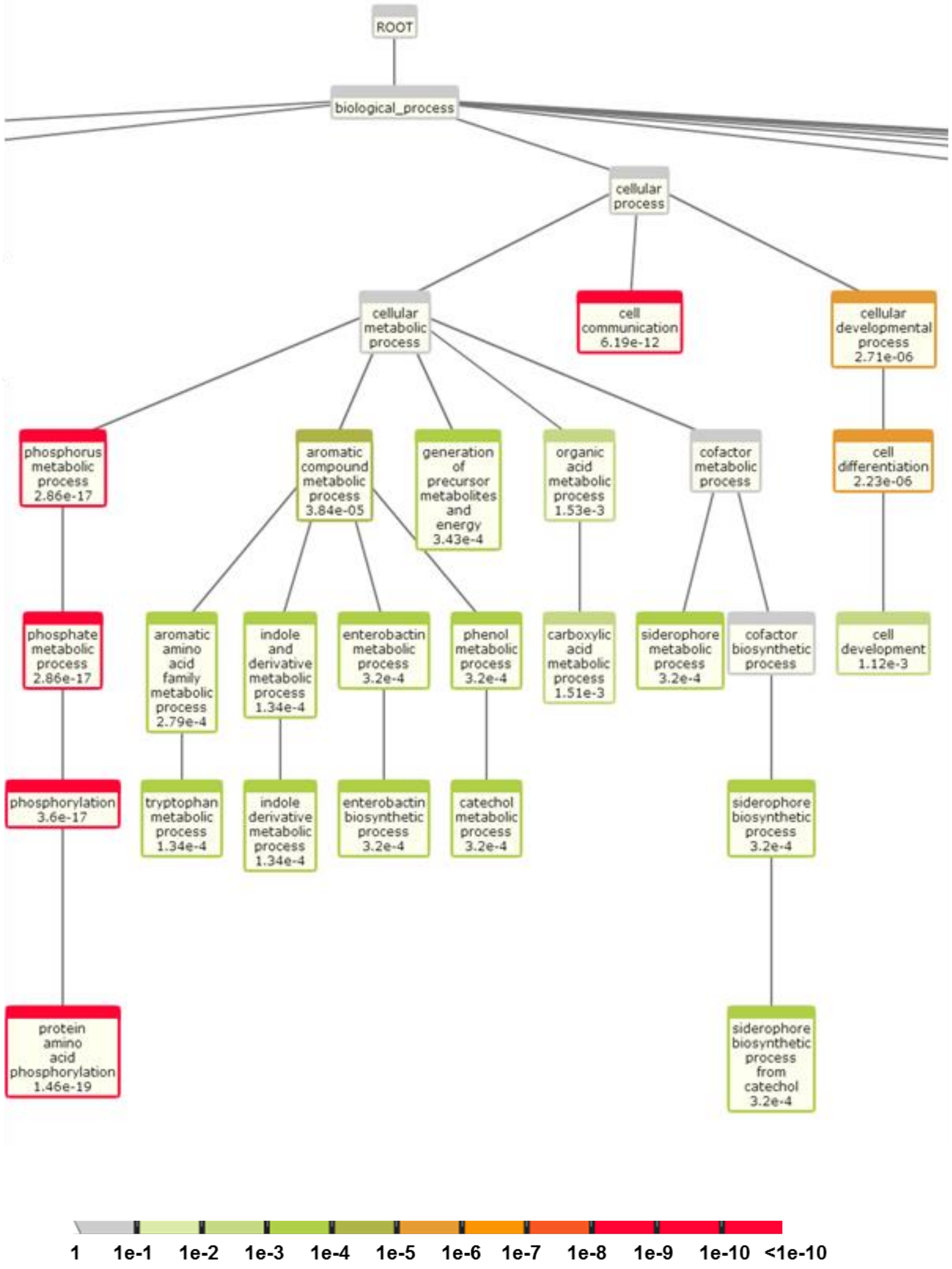
ROOT
biological_process
cellular process
cellular metabolic process
cell communication 6.19e-12
cellular developmental process 2.71e-06
phosphorus metabolic process 2.86e-17
aromatic compound metabolic process 3.84e-05
generation of precursor metabolites and energy 3.43e-4
organic acid metabolic process 1.53e-3
cofactor metabolic process
cell differentiation 2.23e-06
phosphate metabolic process 2.86e-17
aromatic amino acid family metabolic process 2.79e-4
indole and derivative metabolic process 1.34e-4
enterobactin metabolic process 3.2e-4
phenol metabolic process 3.2e-4
carboxylic acid metabolic process 1.51e-3
siderophore metabolic process 3.2e-4
cofactor biosynthetic process
cell development 1.12e-3
phosphorylation 3.6e-17
tryptophan metabolic process 1.34e-4
indole derivative metabolic process 1.34e-4
enterobactin biosynthetic process 3.2e-4
catechol metabolic process 3.2e-4
siderophore biosynthetic process 3.2e-4
protein amino acid phosphorylation 1.46e-19
siderophore biosynthetic process from catechol 3.2e-4
1
1e-1
1e-2
1e-3
1e-4
1e-5
1e-6
1e-7
1e-8
1e-9
1e-10
<1e-10

Figure S6b

C

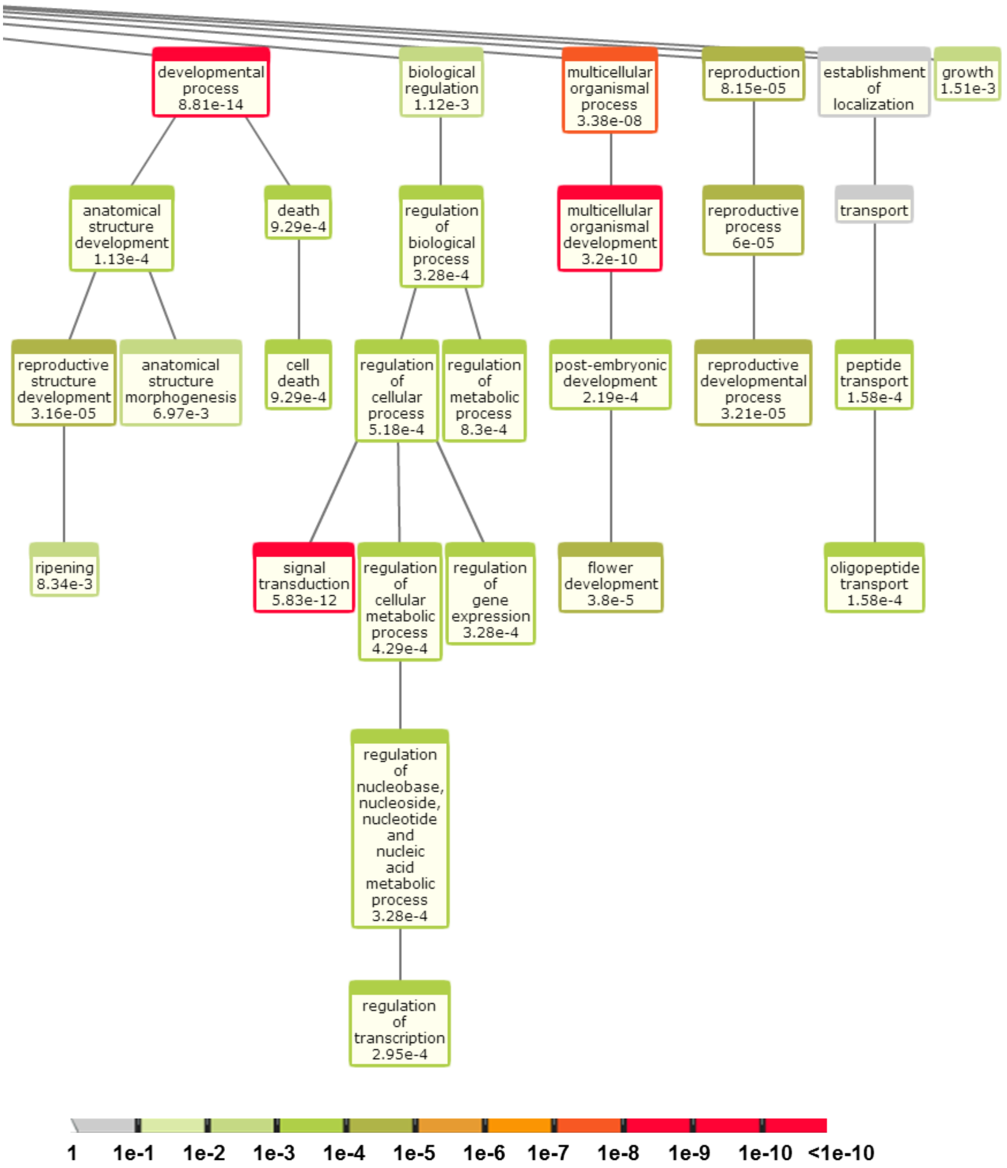

Figure S6: GO categorization of genes induced by cytokinin in the root Using the VirtualPlant (http://virtualplant.bio.nyu.edu) tool for gene ontology (GO) term enrichment, analysis was performed on genes induced by cytokinin in root tissues of rice. (A) The far left-hand side of the network, (B) the mid-section of the network and (C) the far right-hand side of the network. All terms have p-value cutoff of < 0.01 and the p-value of the GO group is depicted by the color scale below.

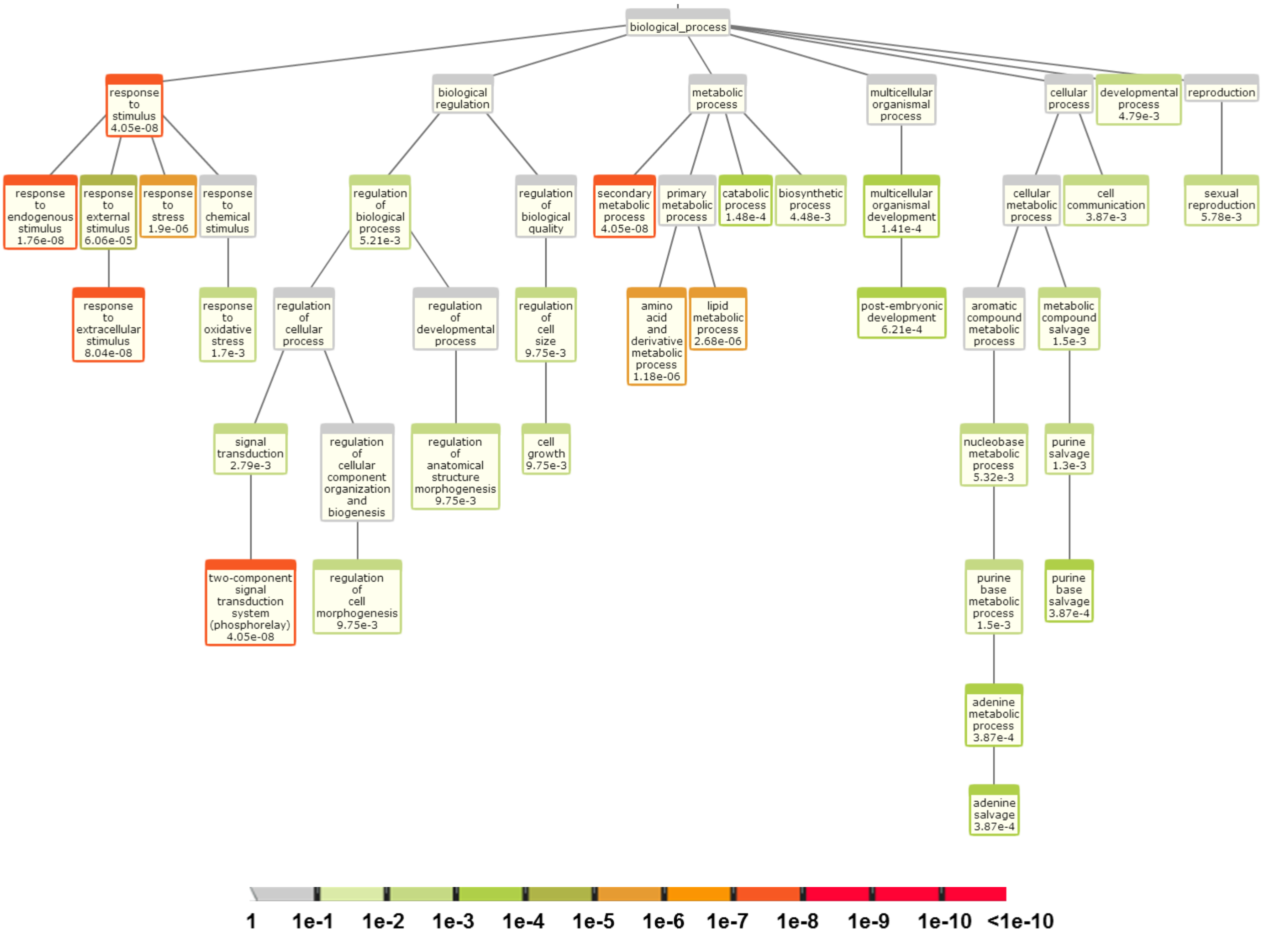

Figure S7: GO categorization of genes induced by cytokinin in the shoot Using the VirtualPlant (http://virtualplant.bio.nyu.edu) tool for gene ontology (GO) term enrichment, analysis was performed on genes induced by cytokinin the shoot tissues of rice. All terms have p-value cutoff of < 0.01 and the p-value of the GO group is depicted by the color scale below.

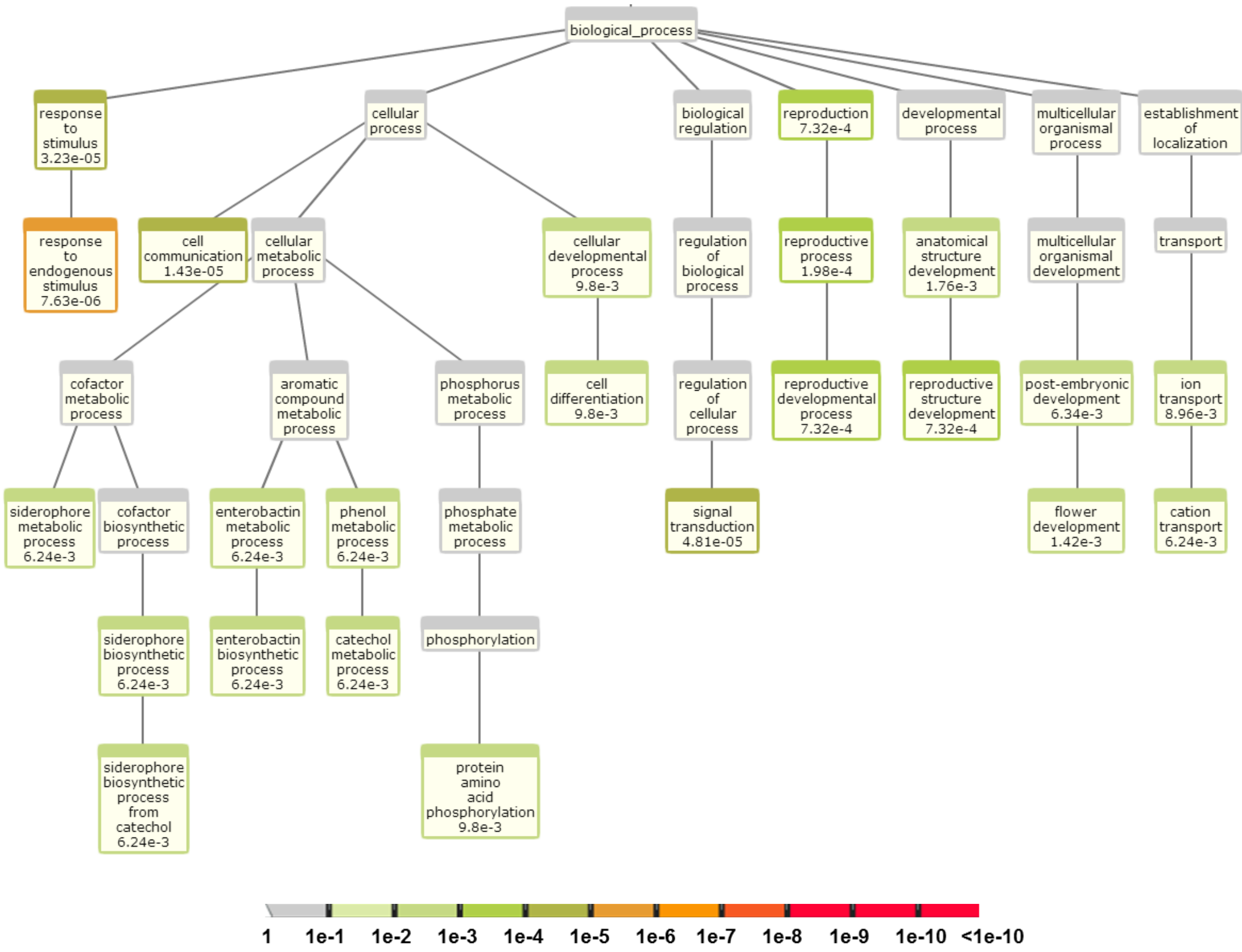

Figure S8: GO categorization of genes repressed by cytokinin in the shoot Using the VirtualPlant (http://virtualplant.bio.nyu.edu) tool for gene ontology (GO) term enrichment, analysis was performed on genes repressed by cytokinin in the shoot tissues of rice. All terms have p-value cutoff of < 0.01 and the p-value of the GO group is depicted by the color scale below.

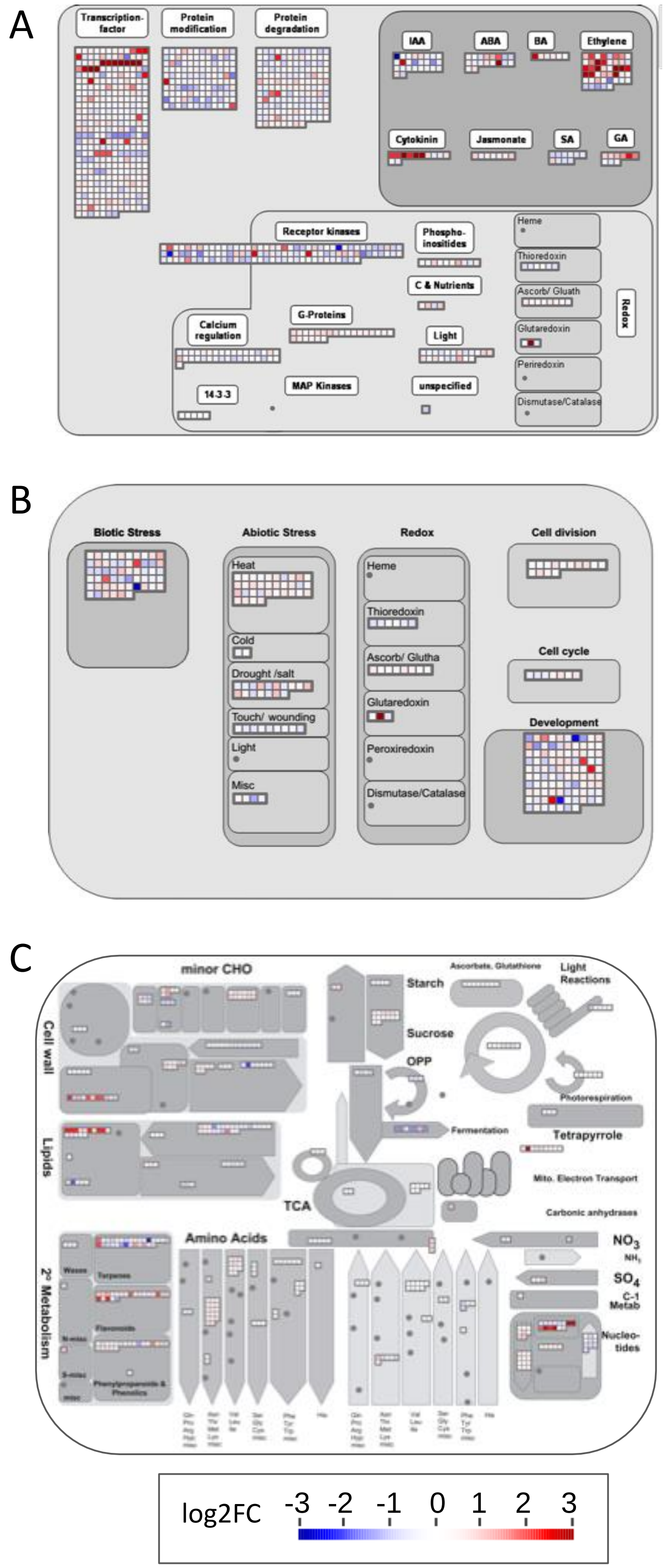

Figure SS9. MapMan overviews of (A) metabolism, (B) cellular response and (C) regulation of cytokinin regulated genes in shoots in rice.
